# L-selectin and Skin Damage in Systemic Sclerosis

**DOI:** 10.1371/journal.pone.0044814

**Published:** 2012-09-13

**Authors:** James V. Dunne, Stephan F. van Eeden, Kevin J. Keen

**Affiliations:** 1 Department of Medicine, University of British Columbia, Vancouver, British Columbia, Canada; 2 British Columbia Scleroderma Clinic, Vancouver, British Columbia, Canada; 3 James Hogg Heart and Lung Institute, Vancouver, British Columbia, Canada; 4 Department of Mathematics and Statistics, University of Northern British Columbia, Prince George, British Columbia, Canada; University of Southern California, United States of America

## Abstract

**Background:**

L-selectin ligands are induced on the endothelium of inflammatory sites. L-selectin expression on neutrophils and monocytes may mediate the primary adhesion of these cells at sites of inflammation by mediating the leukocyte-leukocyte interactions that facilitate their recruitment. L-selectin retains functional activity in its soluble form. Levels of soluble L-selectin have been reported as both elevated and lowered in patients with systemic sclerosis (SSc). This preliminary study seeks to discern amongst these disparate results and to discover whether there is an association between L-selectin concentrations in plasma and skin damage in SSc patients.

**Methodology and Principal Findings:**

Nineteen cases with limited systemic sclerosis (lSSc) and 11 cases with diffuse systemic sclerosis (dSSc) were compared on a pairwise basis to age- and sex-matched controls. Criteria of the American College of Rheumatology were used to diagnose SSc. Skin involvement was assessed using the modified Rodnan skin score (mRSS). We find no association between mRSS and plasma L-selectin concentration in lSSc cases (p = 0.9944) but a statistically significant negative correlation in dSSc cases (R^2^ = 73.11 per cent, p = 0.0008). The interpretation of the slope for dSSc cases is that for each increase of 100 ng/ml in soluble L-selectin concentration, the mRSS drops 4.22 (95 per cent CI: 2.29, 6.16). There was also a highly statistically significant negative correlation between sL-selectin and disease activity (p = 0.0007) and severity (p = 0.0007) in dSSc cases but not in lSSc cases (p = 0.2596, p = 0.7575, respectively).

**Conclusions and Significance:**

No effective treatments exist for skin damage in SSc patients. Nor is there a laboratory alternative to the modified Rodnan skin score as is the case for other organs within the body. Modulation of circulating L-selectin is a promising target for reducing skin damage in dSSc patients. Plasma levels of soluble L-selectin could serve as an outcome measure for dSSc patients in clinical trials.

## Introduction

Systemic sclerosis (SSc) is an inflammatory obliterative microvasculopathy disorder of unknown etiology characterized by excessive collagen deposition causing fibrosis predominantly in the dermis but also in internal organs. Systemic sclerosis is a very rare rheumatic disease that affects about 250 individuals per one million adults and carries the burden of a standardized mortality rate of 3.53 [95% CI: 3.03, 4.11], that is, a survival rate of 70 per cent at 10 years after onset [1]. A hallmark of the disorder is circulating auto-antibodies. It has been proposed that the primary insult is to the microvasculature with activation of the endothelium promoting the recruitment of leukocytes into the extravascular space.

Adhesion molecules borne by both endothelial cells and circulating leukocytes guide the process of extravasation. The selectin family of adhesion molecules is responsible for the early stages of leukocyte adhesion and recruitment involving initial endothelial-leukocyte contact with rolling of the leukocytes on the endothelium. L-selectin (CD62L) is expressed on leukocytes and P- and E-selectin on the endothelium [2]. It has been demonstrated using L-selectin deficient mice that defects in this initial adhesive interaction are responsible for the inability of T cells to home to and be sensitized within peripheral lymph nodes [3]. L-selectin appears to play a critical role in comparison to P- and E-selectin with L-selectin deficient mice essentially equivalent to normal mice treated with antibodies blocking P- and E-selectin [4]. In addition, L-selectin expression on circulating nonspecific effector cells (neutrophils and monocytes) has been suggested to be an important mediator of the primary adhesion of these cells at sites of inflammation [5]. It has been further suggested that L-selectin may exert its effect at inflammatory sites by mediating leukocyte-leukocyte interactions that facilitate the recruitment of neutrophils and lymphocytes [6], [7]. Uniquely, L-selectin is shed after cellular activation, retains functional activity in its soluble form and is involved in the regulation of leukocyte attachment to an inflamed endothelium [8]. Circulating soluble L-selectin (sL-selectin) derives mostly from lymphocytes [7], [9]. L-selectin ligands are induced on the endothelium of inflammatory sites and the cutaneous sites of chronic inflammation [10]. However, sL-selectin may inhibit the attachment of lymphocytes to cytokine-activated endothelium [8].

Decreased levels of sL-selectin have been found in patients with coronary artery disease or acute respiratory distress syndrome compared to controls and it has been postulated that this reflects ongoing endothelial and leukocyte activation [9], [11]. We also report that sL-selectin concentration in plasma was found to be decreased in dSSc cases in comparison to controls but both lSSc cases and controls had similar levels. Levels of sL-selectin have been reported as elevated with concomitant decreased expression on CD8+ cells in SSc [12]. Blann *et al*. had found a lower mean plasma concentration of sL-selectin in 18 SSc patients compared to 42 controls but did not characterize SSc cases as limited (lSSc) or diffuse (dSSc) [13]. Shimada *et al*. reported higher mean sL-selectin levels in plasma in 25 lSSc patients and in 26 dSSc patients compared to their 30 controls [12]. In a murine contact hypersensitivity model, L-selectin was found to mediate infiltration of inflamed skin by CD8+ type 1 cytoxic T (Tc1) cells [14], suggesting that L-selectin is a possible target for therapeutic modulation of skin damage in diffuse cutaneous disease.

The purpose of this preliminary study was to determine in SSc patients, with limited or diffuse disease, whether sL-selectin concentration in plasma is associated with skin damage. The finding of a significant correlation would be a novel result not previously described [15].

## Materials and Methods

### Objectives

Hypothesis tests were conducted to assess whether sL-selectin in plasma was lower in lSSc and dSSc cases compared to their age- and sex-matched controls. Hypothesis tests were conducted to determine the strength of the linear association between plasma levels of sL-selectin and each of disease severity, disease activity and the modified Rodnan skin score (mRSS) in lSSc and dSSc patient groups.

### Participants

Thirty cases with SSc and 30 age- and sex-matched controls participated in this study. All patients were seen at the British Columbia Scleroderma Clinic at St Paul’s Hospital in Vancouver which serves the province of British Columbia which has a universal health care insurance system for its population of 4.4 million. Sequential patients, either new or follow up, from a population of 265 SSc patients were enrolled in the study. None approached refused to participate. Healthy controls were recruited from health care staff, hospital volunteers, or partners of patients. All gave informed written consent in compliance with the Declaration of Helsinki and the Research Ethics Boards of the University of British Columbia and the University of Northern British Columbia. All patients fulfilled the American College of Rheumatology Criteria for SSc and were classified as having limited or diffuse disease according to the criteria of LeRoy *et al*. [16]. None of the age- and sex-matched controls had hypertension, known coronary artery disease, diabetes or lung disease.

Each SSc case was age- and sex-matched with one healthy control. Upon statistical analysis, a mismatch with respect to sex was discovered for one male dSSc case so this case-control pair were dropped from any pairwise comparisons and the healthy control from any statistical analysis. For dSSc cases: one match had an age difference of 7 years, 90% of the other matches with controls were within 4 years. For lSSc cases: 100% were matched within 5 years, 89% of the other matches with controls were within 4 years. Age- and sex-matching were done to minimize as much as possible potentially confounding effects of age and gender in a small sample. The cases considered do form a cohort but in the context of this study, only a cross-sectional snapshot in time is considered.

### Clinical Variables

Disease duration was measured from the onset of the first non-Raynaud’s phenomenon. Skin involvement was assessed using the modified Rodnan skin score at 17 different body areas [17]. Disease severity for each patient was evaluated on the degree of involvement of nine organ systems using the Medsger scale [18] and then summed. Disease activity was scored by the indices developed by Valentini *et al*. in which ten clinical items are given weighted scores on a scale of zero (no activity) to ten (maximal activity) [19]. Active ulcers were documented at the time of phlebotomy and classified as previously described [20], [21]. Pulmonary function was examined but only predicted forced vital capacity (FVC) and predicted diffusing level of carbon monoxide (DLCO) are reported. All clinical variables were recorded at time of plasma collection or within 2 months prior.

In the instance of variables relating to physical assessment by a rheumatologist, these were obtained from clinical record review by the rheumatologist who did the physical assessment. All imaging, lung function and blood test results were obtained from record review by the rheumatologist or pulmonologist who ordered the tests. The pulmonary function tests were conducted by respiratory technicians under the supervision of an MD specialist.

### sL-selectin Concentration

All plasma samples from cases and controls were aliquoted and stored at −80 C until analysis. Plasma levels of sL-selectin were measured using a Searchlight Custom Array (Pierce Searchlight Products, Thermo Fisher Scientific Woburn, Massachusetts, USA) and analyzed using a 16-bit Searchlight CCD imaging and analysis system (Pierce Biotechnology, Rockford, Illinois, USA).

### Ethics Statement

Research was carried out in compliance with the Helsinki Declaration of 1975, as revised in 1983, and approved by the Research Ethics Boards of The University of British Columbia and The University of Northern British Columbia. All participants signed informed consent forms approved by the Research Ethics Board of The University of British Columbia.

### Statistical Analysis

Estimates of location are reported as a mean ± SE. Given small sample sizes, nonparametric methods were used for comparisons. Statistical calculations and graphing were done using release 2.11.1 of the R statistical software system [22]. Normality was assessed by the Shapiro-Wilk test. Welch’s version of the two-sample *t* test was used when homoscedasticity (equal variances) was not assumed. The *stats* package in R was used to calculate the exact *P* values for the Wilcoxon test of no shift in location for matched case-control pairs. The *coin* package in R was used to calculate the exact *P* values for the Mann-Whitney test for location with two independent random samples [23].

## Results

### Demographics and Clinical Features of Patients

Blood samples were available for 19 patients diagnosed with lSSc and 11 with dSSc (Table 1). No gender difference in age was detected for lSSc patients (p = 0.9773) or for dSSc patients (p = 0.6970). Combining genders, patients diagnosed with lSSc had a mean (±SE) age of 58.53±2.50 years (range: 36–74 years) while patients diagnosed with dSSc had a mean age of 49.64±3.17 years (range: 30–65 years). The difference in median age between lSSc and dSSc is statistically significant (p = 0.0399).

**Table 1:** Demographics, auto-antibodies and treatments.

	lSSc		dSSc	
Variable	Estimate	Count	Estimate	Count
Proportion Female:Male	18.0∶1	18∶1	1.2∶1	6∶5
Modified Rodnan Skin Score	9.05±1.29	19	22.82±2.82	11
Severity Index	7.00±0.78	18	7.18±0.91	11
Activity Index	3.39±0.38	18	4.27±0.75	11
Duration of Disease	10.68±2.68 yr	19	2.91±0.48 yr	11
Age				
Cases	58.53±2.50 yr	19	49.64±3.17 yr	11
Controls	59.42±2.53 yr	19	49.80±3.64 yr	10
Lung				
ILD	47.4±11.5%	19	63.6±14.5%	11
PAH	47.4±11.5%	19	36.4±14.5%	11
FVC Predicted	86.6±4.5%	16	73.4±6.5%	11
DLCO Predicted	63.1±4.9%	19	64.8±8.3%	10
Telangiectasia	17.6±9.2%	17	20.0±12.6%	10
Digits				
Raynaud’s	100%	18	100%	11
Active digital ulcers	35.3±11.6%	17	30.0±14.5%	10
No. of digits with active digits ulcers	0.71±0.28	17	0.88±0.48	9
Contracture of phalanges	41.2±11.9%	17	63.6±14.5%	11
Autoantibodies				
ACA positive	15.8±8.4%	19	0%	11
ANA positive	84.2±8.4%	19	90.1±8.7%	11
Scl-70 positive	15.8±8.4%	19	72.7±13.4%	11
Treatment				
Methotrexate	0%	19	18.2±11.6%	11
Cyclophosphamide	0%	19	45.5±15.0%	11

Values given as mean ± SE or proportion ± SE if a percentage is indicated.

No gender difference in disease duration was detected for lSSc (p = 0.6895) or for dSSc (p = 0.8485) patients. Combining genders, patients diagnosed with lSSc had a mean (±SE) disease duration of 10.68±2.68 years (range: 1–43 years) whilst patients diagnosed with dSSc had a mean disease duration of 2.91±0.48 years (range: 2–11 years). Disease duration is significantly shorter (p = 0.0381) in dSSc cases compared to lSSc as expected.

A gender difference in mRSS was detected between the 18 women and the sole man for lSSc (p = 0.0004) but not amongst men and women for dSSc (p = 0.7576). Mean (±SE) mRSS was 9.05±1.29 (range: 1–20) in lSSc cases and 22.82±2.82 (range: 4–36) in dSSc cases. Skin score is worse in dSSc cases compared to lSSc as expected (p = 0.0002).

There was no difference in location of the distribution of disease severity scores between women and the single man with lSSc (p = 0.2774) or amongst the female and male dSSc patients (p>0.9999). Combining genders, patients diagnosed with lSSc had a mean (±SE) disease severity index of 7.00±0.78 (range: 2–16). Patients diagnosed with dSSc had a mean maximum disease severity index of 7.18±0.91 (range: 2–11).

There was a difference in location of the distribution of activity scores between the 18 women and the single man with lSSc (p = 0.0033) but not amongst the female and male dSSc patients (p = 0.7662). Combining genders, patients diagnosed with lSSc had a mean (±SE) activity index of 3.39±0.38 (range: 1–18). Patients diagnosed with dSSc had a mean activity index of 4.27±0.75 (range: 1–8).

There was no statistically significant difference between lSSc and dSSc cases with respect to the location of the distribution of the disease severity index (*p* = 0.6638). Likewise, there was no difference between lSSc and dSSc cases with respect to the location of the distribution of the disease activity index (*p* = 0.7662). It is speculated that the lack difference in disease severity and activity between the limited and diffuse forms of the disease are a consequence of either aggressive clinical management or referral bias, or both, in this population of patients being treated in a tertiary scleroderma clinic serving 4.4 million people in the province of British Columbia, Canada.

On the basis of the preceding statistical analyses, decisions were made to disregard gender but treat limited and diffuse forms of SSc as distinct.

With regard to autoantibodies and treatment modalities, dSSc and lSSc cases have distinctly different profiles (Table 1).

### mRSS, Severity and Activity

**Figure 1:**
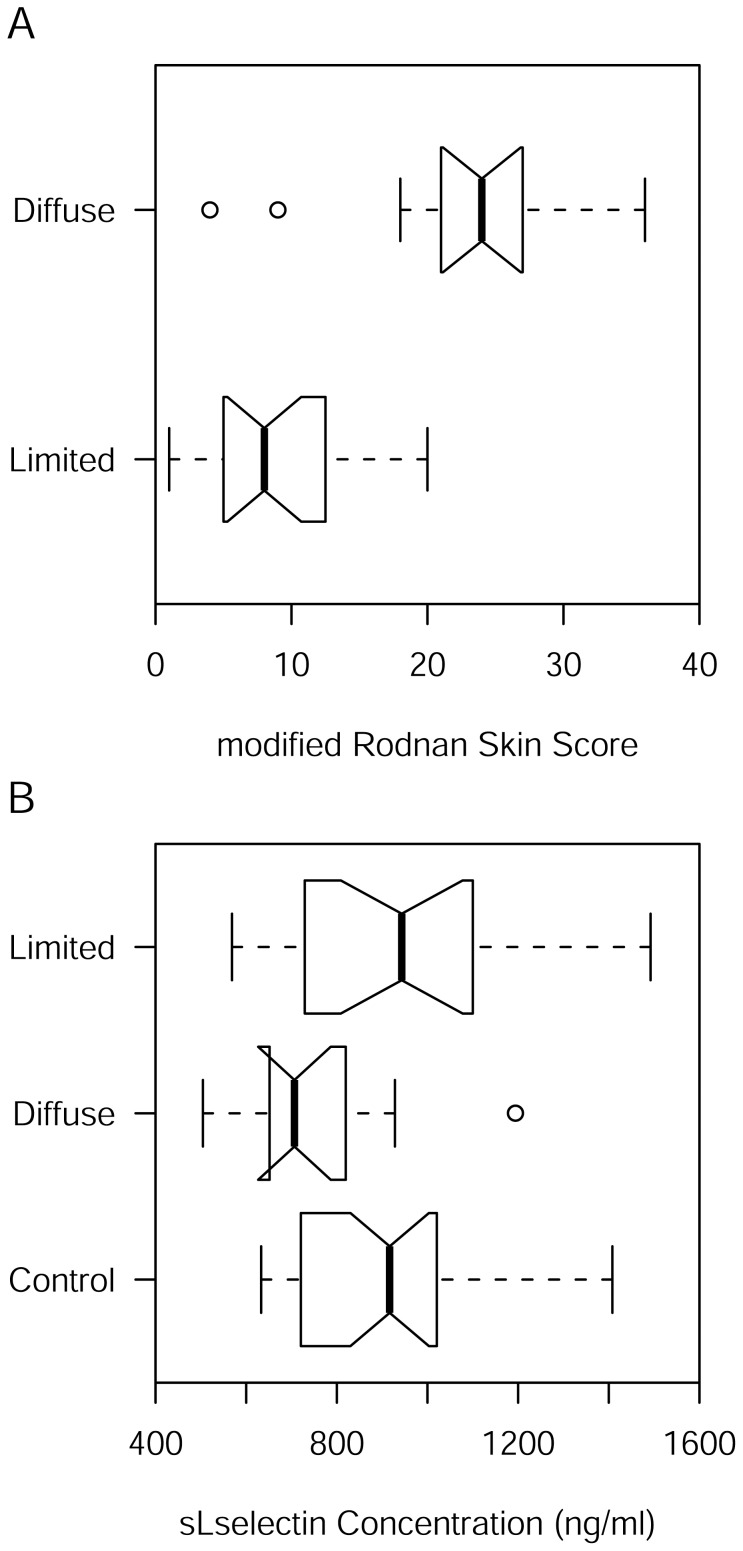
Notched boxplots of mRSS and sL-selectin. If the notches overlap in a pairwise comparison then the difference is significant at the 5% level for the individual test. (A) Notched boxplot of mRSS. (B) Notched boxplot of sL-selectin.

The distribution of mRSS appears to be normal for both lSSc (p = 0.3300) and dSSc (p = 0.2318) cases. Assuming normality, the estimate of the mean for mRSS is 9.05 (95 per cent CI: 6.35, 11.76) and 22.82 (95 per cent CI: 16.52, 29.11) in lSSc and dSSc cases, respectively. As expected, the means for lSSc and dSSc cases are significantly different by Welch’s two-sample *t* test (p = 0.0005, 95 per cent CI: 7.12, 20.41). In fact, mean mRSS is significantly lower in lSSc cases compared to dSSc cases (Figure 1 A).

The distribution of the disease severity index appears to be normal for both lSSc (p = 0.2490) and dSSc (p = 0.6222). Without assuming equal variances, the means are not different by Welch’s two-sample *t* test (p = 0.8812, 95 per cent CI: −2.31, 2.67).

The distribution of the disease activity index appears to be normal for both lSSc (p = 0.5294) and dSSc (p = 0.9512). Without assuming equal variances, the means are not different by Welch’s two-sample *t* test (p = 0.3086, 95 per cent CI: −0.90, 2.67).

### sL-selectin Concentration

The distribution of sL-selectin concentration appears to be normal for controls (p = 0.1329), lSSc (p = 0.4153) and dSSc (p = 0.3657) cases. The point estimate of the mean sL-selectin concentration is 906 (95 per cent CI: 834, 978) ng/ml in controls. The point estimate of the mean sL-selectin concentration is 961 (95 per cent CI: 833, 1090) ng/ml and 755 (95 per cent CI: 628, 882) ng/ml in lSSc and dSSc cases, respectively. Because sL-selectin concentration was measured in controls as well as cases, it is possible to test the null hypothesis of no location shift between cases with pairwise matched controls. One male dSSc case did not match a female control and so both were dropped from comparison for sL-selectin. Because the number of pairs is 19 for lSSc and only 10 for dSSc, it would be prudent to use the nonparametric Wilcoxon signed rank test instead of the matched pairs *t* test. There appears to be no shift of location for lSSc cases (p = 0.2753) but there does appear to be a shift for dSSc cases (p = 0.0273). Nevertheless, and consistently, a paired *t* test for the mean difference for lSSc cases compared to their matched controls is statistically insignificant (p = 0.2424, 95 per cent CI: −62, 229 ng/ml) while the mean difference for dSSc cases compared to their matched controls is statistically significant (p = 0.0369, 95 per cent CI: −362, −14 ng/ml). The mean sL-selectin concentrations for lSSc and dSSc cases are significantly different by Welch’s two-sample *t* test (p = 0.0005, 95 per cent CI: 7, 20 ng/ml). So we can conclude that the mean sL-selectin concentration for dSSc is lower than that of either lSSc cases or controls (Figure 1 B).

### Association between sL-selectin Concentration and mRSS

The standard test assuming normality for a linear relationship between mRSS and sL-selectin concentration in lSSc cases is statistically insignificant (0.9944). The point estimate of the simple correlation coefficient is 0.00172 with 95 per cent confidence interval (−0.45284,.45558). The point estimate of Spearman’s rho, the nonparametric counterpart, is −0.0026 (p = 0.9914).

The standard test assuming normality for a linear relationship between mRSS and sL-selectin concentration in dSSc cases is highly statistically significant (p = 0.0008). The point estimate of the simple correlation coefficient is −0.855021 with 95 per cent confidence interval (−0.96166, -.52381). The point estimate of Spearman’s rho, the nonparametric counterpart, is −0.72189 (p = 0.0121).

*P*-values for tests of normality for the residuals of the simple linear regression model for mRSS as a function of sL-selectin concentration are 0.3318 and 0.5165 for lSSc and dSSc cases, respectively. Thus there is no need to resort to the nonparametric alternative of Spearman’s rho.

**Figure 2:**
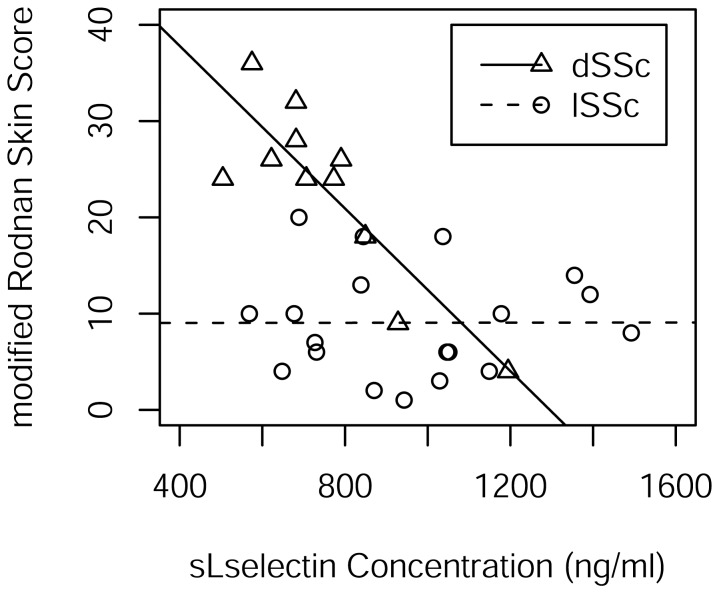
Scatterplot of mRSS and sL-selectin for lSSc and dSSc cases. There are separate least squares regression lines for each of lSSc and dSSc cases.

We conclude that the separate simple linear regression models for each of lSSc and dSSc cases fit the data well (Figure 2). The slope of the regression line for lSSc cases is not different from zero (p = 0.9944). The estimate of the slope of the regression line for dSSc is (−0.04224, 95 per cent CI: −0.06156, −0.02292 (ng/ml) ^−1^).

**Figure 3:**
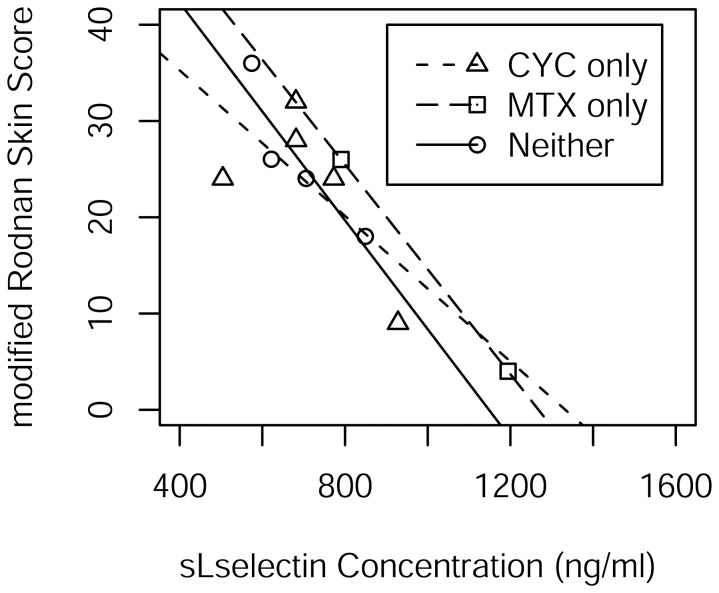
Scatterplot of mRSS and sL-selectin indicating drug prescribed for dSSc cases only. There are separate least squares regression lines for dSSc cases receiving cyclophosphamide (CYC) only, methotrexate (MTX) only, and neither.

Cyclophosphamide (CYC) has been implicated in the reduction of CD34+ cells expressing L-selectin [24]. Likewise, the combination of CYC, methotrexate (MTX) and an infusion of CD62L+ T cells expressing L-selectin has been associated with improved disease-free and overall survival in patients receiving allogeneic hematopoietic stem cell transplants [25]. As 6 of the 11 dSSc patients were receiving CYC and another 2 were receiving MTX, hypothesis tests were conducted separately for the inclusion of CYC status and MTX status with sL-selectin in the linear model for mRSS (Figure 3). The tests for CYC (p = 0.7652) and MTX (p = 0.6795) were negative for inclusion as was the test for inclusion of both (p = 0.8950).

To allay concerns about a dosage-duration effect, we did a chart review and found with one exception that all dSSc patients who had been on either cyclophosphamide or methotrexate were on it for 2 years or less and maintained high mRSS scores. The one exception was the patient in Figure 3 with the second lowest modified Rodnan skin score who had been on oral cyclosphosphamide for 4 years and maintained a low score for the two years prior to testing from an initial score of 30. Running a test whereby this patient is dropped from the simple linear regression analysis, we find the estimate of Pearson’s correlation coefficient to be −0.85045 (P = 0.00182) and Spearman’s rank correlation to be −0.62767 (p = 0.05198). With the patient included, the estimate of Pearson’s correlation coefficient is −0.855021 (p = 0.0008) and Spearman’s rank correlation is −0.72189 (p = 0.0121). In short, there is not much of a difference with or without the patient.

### Association between sL-selectin Concentration and Disease Severity

The standard test assuming normality for a linear relationship between disease severity and sL-selectin concentration in lSSc cases is statistically insignificant (p = 0.2596). The point estimate of the simple correlation coefficient is −0.28048 with 95 per cent confidence interval (−0.66082, 0.21448). The point estimate of Spearman’s rho, the nonparametric counterpart, is −0.3738 (p = 0.1265).

The standard test assuming normality for a linear relationship between disease severity and sL-selectin concentration in dSSc cases is highly statistically significant (p = 0.0007). The point estimate of the simple correlation coefficient is −0.85795 with 95 per cent confidence interval (−0.96247, −0.53174). The point estimate of Spearman’s rho, the nonparametric counterpart, is −0.7717 (p = 0.0054).

P-values for tests of normality for the residuals of the simple linear regression model for disease severity as a function of sL-selectin concentration are 0.3396 and 0.5167 for lSSc and dSSc cases, respectively. Thus there is no need to resort to the nonparametric alternative of Spearman’s rho.

We conclude that the separate simple linear regression models for each of lSSc and dSSc cases fit the data well. The slope of the regression line for lSSc cases is not different from zero (p = 0.2596). The estimate of the slope of the regression line for dSSc is (−0.01370, 95 per cent CI: −0.01988, −0.00751 (ng/ml) ^−1^).

### Association between sL-selectin Concentration and Disease Activity

The standard test assuming normality for a linear relationship between disease activity and sL-selectin concentration in lSSc cases is statistically insignificant (p = 0.7575). The point estimate of the simple correlation coefficient is −0.07828 with 95 per cent confidence interval (−0.52593, 0.40333). The point estimate of Spearman’s rho, the nonparametric counterpart, is −0.20313 (p = 0.4188).

The standard test assuming normality for a linear relationship between activity and sL-selectin concentration in dSSc cases is highly statistically significant (p = 0.0007). The point estimate of the simple correlation coefficient is −0.85897 with 95 per cent confidence interval (−0.96276, −0.53451). The point estimate of Spearman’s rho, the nonparametric counterpart, is −0.87416 (p = 0.0004).

*P*-values for tests of normality for the residuals of the simple linear regression model for severity as a function of sL-selectin concentration are 0.5177 and 0.4891 for lSSc and dSSc cases, respectively. Thus there is no need to resort to the nonparametric alternative of Spearman’s rho.

We conclude that the separate simple linear regression models for each of lSSc and dSSc cases fit the data well. The slope of the regression line for lSSc cases is not different from zero (p = 0.0071). The estimate of the slope of the regression line for dSSc is (−0.01125, 95 per cent CI: −0.01631, −0.00619 (ng/ml) ^−1^).

## Discussion

We found no statistically significant linear association between sL-selectin concentration and either of disease severity (p = 0.2596), disease activity (p = 0.7575) or mRSS (p = 0.9944) in lSSc cases. But we did find statistically significant associations in dSSc cases between sL-selectin concentration and each of disease severity (p = 0.0007), activity (p = 0.0007) and mRSS (p = 0.0008). The amount of variation in dSSc cases for each of disease severity, disease activity, and mRSS explained by a linear relationship with sL-selectin is 73.61 per cent, 73.78 per cent and 73.11 per cent, respectively. The amount of variation explained by the linear association with sL-selectin is arguably the lowest for mRSS amongst the three variables but we have chosen to focus in this discussion on the skin score mRSS because in part the other two indices are functions of mRSS. The definition for disease activity adds 0.5 to a maximum score of 10 if mRSS exceeds 14 [19], whereas, that for disease severity has a graded approach with zero (mRSS = 0), 1 (1 ≤ mRSS ≤ 14), 2 (15 ≤ mRSS ≤ 29), 3 (30 ≤ mRSS ≤ 39) and 4 (mRSS ≥ 40) [18]. Moreover, it can be argued that either the disease activity index or disease severity score capture only an additional 0.67 and 0.5 per cent, respectively, of variation for all the other organs combined. Disease activity has additional variables for skin but also for the heart, lungs, muscles, kidneys, and vasculature. Disease severity assesses these and the gastrointestinal tract too. There is also the issue of no statistically significant difference in mean disease severity and mean disease activity between lSSc and dSSc cases (p = 0.8812, p = 0.3086, respectively) but a striking statistically significant difference in mean mRSS (p = 0.0005) in this group of patients from a tertiary referral facility for scleroderma patients.

We found that sL-selectin levels are low in dSSc cases compared to their controls and within the range of normal controls for lSSc cases. We showed a significant negative relationship between sL-selectin and the magnitude of skin involvement in dSSc. This suggests that sL-selectin could serve as a blood biomarker of the extent of skin involvement in dSSc. It also implicates a possible pathogenic role for L-selectin in the chronic inflammatory obliterative microvasculopathy of dSSc.

Blann *et al*. found a lower mean (±SE) plasma concentration of sL-selectin of 797±71 ng/ml in 18 SSc patients compared to 1 244±42 ng/ml in 42 controls (*P* ≤ 0.0001) but did not characterize SSc cases as limited or diffuse [13]. The results of Blann *et al.* are comparable to our estimates of the mean plasma concentration of soluble serum sL-selectin in dSSc cases (p = 0.6498) and for lSSc cases (p = 0.0890) but not our controls (p<0.0001) (Table 1). However, caution in interpreting these hypothesis tests must be exercised because of the difference in assay methods.

Shimada *et al*. reported higher mean (±SE) sL-selectin serum levels in 25 lSSc patients (1 280±130 ng/ml) and in 26 dSSc patients (1 830±324 ng/ml) compared to their 30 controls (990±450 ng/ml) [12]. The differences between Shimada *et al*. mean values and ours are unclear but may be due to different antibodies and techniques used to measure sL-selectin. Inaoki *et al*. also reported significantly (p<0.0100) higher mean (±SE) sL-selectin serum levels in 24 SSc patients (2 260±1590 ng/ml) compared to 20 age- and sex-matched controls (1 200±400 ng/ml) [26]. Sato *et al*. similarly reported significantly (0.0100<p<0.0500) higher mean (±SE) sL-selectin serum levels in 32 SSc patients (1 500±1300 ng/ml) compared to 20 age- and sex-matched controls (900±400 ng/ml) [15]. The articles by Shimada *et al*., Inaoki *et al*., and Sato *et al*. were all published in 2001 from the same laboratory at Kanazawa University in Japan using the same assay. As the counts of cases and controls are not equal in each of these three articles, it is not know how the matching was done. As it is possible that there is an overlap in cases for the three articles, a meta analysis is not possible. Spertini *et al*. showed no difference in the concentration of sL-selectin measured in serum as compared to plasma for the assay method of the three papers from the laboratory at Kanazawa University [27]. Differences in assay, however, has no bearing on the finding in these three articles of significantly higher levels of sL-selectin in serum in SSc cases compared to healthy controls. This is just the opposite of findings by Blann *et al*. and this study. Neither Sfikakis *et al*. [28] or Ates *et al*. [29] found a significant difference in the location of serum sL-selectin levels between cases and controls but neither study differentiated between lSSc and dSSc.

All studies are limited by being cross-sectional on a small number of patients. Clearly larger sample sizes are necessary.

A major point of departure for this study compared to its predecessors is the examination of the relationship between the concentration of soluble L-selectin and the modified Rodnan skin score in lSSc and dSSc cases, separately. Blann *et al*. [13], Shimada *et al*. [12], Inaoki *et al.* [26], Sfikakis *et al*. [28], and Ates *et al*. [29] did not examine the relationship between the concentration of soluble L-selectin and the modified Rodnan skin score. Incidental to their report of interleukin-6 and interleukin-10 correlating with mRSS, Sato *et al*. [15] stated: “Furthermore, multiple regression analysis showed that modified Rodnan TSS was significantly positively correlated with serum IL-6 levels (P<0.05) and IL-10 levels (P<0.005), but not with other soluble mediators.” Presumably, Sato *et al*. did not estimate correlation coefficients for their 16 lSSc and 16 dSSc cases separately. Additionally, Sato *et al*. did not explicitly report an estimate of the correlation coefficient between mRSS and sL-selection serum concentrations in the pooled lSSc and dSSc patients.

This preliminary study has more lSSc cases than the number of SSc cases in Blann *et al*. [13]. It has more lSSc cases than either Sfikakis *et al*. [28], Shimada *et al*. [12], Inaoki *et al.* [26], or Sato *et al*. [15]. This study has as many SSc cases as Ates *et al*. [29] but this study differentiates between limited and diffuse forms of systemic sclerosis while Ates *et al*. [29] did not. There are more controls in this study than either Shimada *et al*. [12], Inaoki *et al.* [26], Sato *et al*. [15]. or Ates *et al*. [29].

In a contact-hypersensitivity murine model, L-selectin deficient mice exhibit a reduction of Tc1 infiltration in inflamed skin compared to wild type [14]. As well, a bleomycin-induced murine model of SSc demonstrated that Th2 and Th17 cell infiltration into skin and lungs was inhibited by L-selectin while L-selectin loss attenuated the development of fibrosis [30]. In a tight skin (L-selectin −/− TSK+) mouse model, however, skin thickness was similar to the skin thickness of TSK+ wild type mice [31]. DNA microarray studies of cutaneous gene expression in a murine sclerodermatous graft-versus-host disease model show mRNA for cell adhesion molecules are upregulated. L-selection, vascular adhesion molecule-1 and intercelleular adhesion molecule-1 are upregulated during the early inflammatory phase of cutaneous fibrosis [32]. This suggests that interference with the selectins and their ligands may be useful candidates for treatment of SSc beginning with dSSc cases.

Soluble L-selectin is generated by the shedding of the L-selectin molecule from the surfaces of neutrophil granulocytes and lymphocytes. It has been reported in a study done in Japan that while the frequency of expression on L-selectin on CD8^+^ T cells was insignificantly different (p>0.0500) in the peripheral blood of 14 SSc cases compared with to 18 health controls, the frequency of expression of L-selectin on the CD161^+^CD8^+^ subset of T cells was significantly increased (p<0.0100) in SSc cases compared to health controls [33]. But the frequency and total number of CD161^+^CD8^+^ T cells in SSc cases was decreased significantly (p<0.0500, p<0.0010, respectively) compared to healthy controls so it is unclear as to whether L-selectin concentration from T cell sources would increase or decrease in SSc cases compared to health controls based on this study from Japan.

Adverse outcomes and posttraumatic complications after surgical trauma have been associated with low levels of sL-selectin and decreased L-selectin expression. Stimulation with tumor necrosis factor alpha (TNF-α) has been shown to effect a significant decrease in L-selectin expression [34]. Taking the diffuse cutaneous form of SSc as a chronic inflammatory disease suggests a possible therapeutic role of a TNF-α blocker, or other biologics, or interference with the selectins or their ligands, in reducing skin damage in SSc patients through the effect of increased L-selectin expression, which can in turn be monitored by looking for increasing soluble L-selectin in the plasma of patients. Elevated serum levels of sL-selectin have been found in SSc patients after intravenous injection of lipo-postaglandin E_1_ which is known to have anti-inflammatory effects on endothelial cells and subsets of leukocytes [26]. Candidates for TNF-α inhibitors include the circulating receptor fusion protein etanercept and the monoclonal antibodies rituximab, infliximab, adalimumab, certolizumab pegol, and golimumab. Although a systematic literature review has shown promising results in small-enrolment clinical trials with respect to histological skin changes for rituximab [35], systematic literature reviews have not revealed consistent statistically significant results for skin score for etanercept, rituximab or infliximab in small-enrolment clinical trials [35], [36]. A clinical study of 72 SSc cases receiving rituximab that were recruited from 27 European centers showed significant improvements in mRSS (p = 0.0002) after 7 months on the drugs, total number of digital ulcers (p = 0.0086), creatine kinase levels (p = 0.0300) and the European SSc activity score (p = 0.0100) but not lung fibrosis, DAS-28, or S-HAQ [37]. This is promising but larger multi-center randomized controlled trials with adequate power are needed before the book is closed on TNF-α blockers, or other treatment modalities, in the context of skin damage in SSc [38].

We conclude that sL-selectin is decreased in subjects with dSSc and showed a negative correlation with the magnitude of skin disease (mRSS). Moreover in dSSc, for each increase of 100 ng/ml in sL-selectin concentration, the modified Rodnan skin score drops 4.22 (95 per cent CI: 2.29, 6.16). Disease severity and disease activity in dSSc cases drop 1.37 (95 per cent CI: 0.75, 1.99) and 1.13 (95 per cent CI: 0.062, 1.63), respectively, for each increase of 100 ng/ml in sL-selectin concentration. The theoretical range in values for disease severity, disease activity, and mRSS are [0, 36], [0, 10] and [0, 51], respectively, while the observed ranges of our sample of dSSc cases were [2], [11], [0.5, 8] and [4], [36], respectively. Thus the drops in disease severity, disease activity and mRSS in dSSc cases for an increase of 100 ng/ml in sL-selectin concentration for disease severity disease activity and mRSS are comparable to a good approximation and in concordance with the postulate that improvement in the skin score is the driving force.

Serum levels of sL-selectin have been shown to increase in acute inflammatory conditions while those with chronic disease have been shown to have low levels suggesting an important role for sL-selectin in modulating chronic inflammatory diseases [9]. The key significance of the finding of a significant negative correlation between mRSS and sL-selectin concentration in the plasma of dSSc patients is the suggestion that *in vivo* modulation of L-selectin, or its ligands, is a pathway for reducing collagen deposition, fibrosis, and avascularization in the skin (the body’s largest organ) in the most desperate of patients with systemic sclerosis, that is, those patients with the diffuse form of the disease. Modulating sL-selectin thus presents a potential therapeutic target for addressing skin damage in dSSc patients.

### Limitations

While comparable with the published literature for SSc, this study is limited by the small samples of lSSc and dSSc cases. There were two independent sets of multiple hypothesis tests for each of lSSc and dSSc. For an overall 0.05 level of significance, a conservative Bonferroni correction would thus be a level of 0.05÷26 = 0.0019 for each individual test. This level was exceeded for the tests of linear association assuming normality between sL-selectin and each of mRSS (p = 0.0008), disease severity (p = 0.0007) and activity (p = 0.0007) for the dSSc cases only.

### Future Research

While participant recruitment continues at the British Columbia Scleroderma Clinic to increase sample size for the purpose of improving the power of hypothesis tests used herein, a proposal for a prospective international multi-center study, with a protocol common to all centers, is being developed.
